# Respiratory syncytial virus preventives for children in Australia: current landscape and future directions

**DOI:** 10.5694/mja2.52671

**Published:** 2025-05-25

**Authors:** Sam T Barnett, Jane Tuckerman, Ian G Barr, Nigel W Crawford, Danielle F Wurzel

**Affiliations:** ^1^ Monash University Melbourne VIC; ^2^ University of Melbourne Melbourne VIC; ^3^ Murdoch Children's Research Institute and The Royal Children's Hospital Melbourne VIC; ^4^ WHO Collaborating Centre for Reference and Research on Influenza Melbourne VIC; ^5^ The Peter Doherty Institute for Infection and Immunity, University of Melbourne Melbourne VIC

**Keywords:** Asthma, Pediatric emergency medicine, Neonatology, Infancy, Vaccine preventable disease, Respiratory tract infections, Childhood diseases, Fetomaternal medicine, Virus diseases

## Abstract

- Respiratory syncytial virus (RSV) is a major cause of acute lower respiratory tract infections, and a leading cause of hospitalisation in children under 6 months of age.
- Previously, palivizumab, a costly, short‐acting monoclonal antibody, was the primary preventive option. The recent introductions of nirsevimab (Beyfortus), a long‐acting monoclonal antibody, and Abrysvo, a maternal RSV vaccine, have brought about significant advances in RSV prevention for children.
- Western Australia, Queensland and New South Wales launched state‐managed nirsevimab programs targeting infants and high risk groups for the 2024 RSV season.
- International data support nirsevimab's effectiveness in reducing RSV‐related hospitalisations and severity of disease in real‐world settings.
- In 2025, Australia's national RSV prevention program includes free maternal vaccination with Abrysvo and targeted infant protection with nirsevimab for high risk or newborns whose mothers did not receive Abrysvo at least 2 weeks before delivery, funded by individual jurisdictions.
- Real‐world efficacy data derived from Australian states and territories and the national prevention program will be pivotal in evaluating and refining the integration of maternal immunisation with Abrysvo and infant passive immunisation with nirsevimab.
- Key logistical considerations include ensuring timely access and equitable distribution, particularly for First Nations populations who face increased risk from RSV infection. Coordinated efforts are essential to overcome health care disparities and deliver effective prevention strategies to these prioritised groups.

Globally, respiratory syncytial virus (RSV) is responsible for an estimated 33 million acute lower respiratory tract infections, resulting in 26 300 in‐hospital deaths annually in children aged 0 to 60 months.^1^ Children under the age of 4 years are disproportionately impacted and represent 50% of cases, with infants under the age of 6 months accounting for most of the RSV‐associated hospitalisations in Australia.^2^

The majority of infants hospitalised with severe RSV infection are otherwise healthy; however, certain risk factors have been identified including: younger age, prematurity, and underlying lung or heart conditions.^3^ First Nations children, who are at an increased risk of lung diseases (eg, asthma and bronchiectasis) compared with non‐First Nations children, also experience higher rates of RSV hospitalisation, with 789 per 100 000 affected in individuals under 5 years, nearly double the rate in non‐First Nations children (420 per 100 000).^4^, ^5^, ^6^, ^7^

Before 2024, the short‐acting monoclonal antibody palivizumab (Synagis, Abbott Laboratories) was the only available RSV preventive in Australia for children up to 2 years of age.^8^ However, the high cost of palivizumab, and the need for monthly injections, limited its use to a small subgroup of infants deemed to be at increased risk of severe RSV infection.^9^ Further, a Western Australian study found that compliance with recommended guidelines and dosing schedules was limited.^10^ On 24 November 2023, nirsevimab (Beyfortus, Sanofi‐Aventis and AstraZeneca), a long‐acting monoclonal antibody, was registered with the Therapeutic Goods Administration (TGA) for prevention of RSV‐related infections in children younger than 2 years of age.^11^ On 20 March 2024, Abrysvo (Pfizer), a maternal RSV vaccine, was also registered with the TGA for the prevention of RSV‐related infections in infants from birth to 6 months of age.^12^ The availability of these two preventives has marked an era‐defining advancement in RSV prevention.

In March 2024, Western Australia was the first Australian jurisdiction to announce a state‐wide government‐funded all‐infant RSV immunisation program. From 1 April 2024 to 30 September 2024, all infants entering their first RSV season (born on or after 1 October 2023) as well as children (aged 8 to 19 months) at increased risk of severe RSV were eligible to receive nirsevimab.^13^ A similar program was adopted in Queensland,^14^ whereas New South Wales opted for a program to only protect infants at increased risk.^15^ Recent data indicate that these programs were associated with reduced hospitalisations and medically attended RSV infections,^16^, ^17^ although ongoing evaluation is needed to determine their broader impact. In November 2024, the Australian Government announced that the maternal RSV vaccine would be available from 3 February 2025 to all pregnant women for free under the National Immunisation Program (NIP).^18^ In parallel, Australian jurisdictions have targeted RSV monoclonal antibody (nirsevimab) programs offered at no cost to high risk or unvaccinated newborns.^18^ Ongoing evaluations of the national RSV prevention program will focus on assessing its impact, cost‐effectiveness and areas for refinement.

A decision to implement RSV preventive strategies involves consideration of multiple factors including timing, logistics, efficacy, coverage and the availability of new preventives. The aim of this narrative review was to critically examine and evaluate current RSV preventive options and prevention strategies to protect infants against RSV in Australia, while also considering their implementation across diverse populations based on health care infrastructure, access and attitudes.

## Methods

The literature for this article draws from diverse sources, including clinical trial data, observational studies, government health reports, press releases and websites. No systematic search strategy was employed; instead, relevant literature was identified through existing expertise and manual searches of key sources. We prioritised studies from 2023–2024 as they provided both experimental and real‐world data on the efficacy and safety of RSV preventives. Grey literature that provided insights into the socio‐economic and health care impacts of RSV prevention programs was included to offer a comprehensive understanding of the implications for public health policy in Australia.

## Short‐acting RSV monoclonal antibody (palivizumab)

Before nirsevimab, palivizumab was the only available RSV immunoprophylaxis in Australia, reserved for population groups at increased risk of severe RSV infection. Clinical trials for palivizumab in infants demonstrated moderate efficacy at preventing RSV‐related hospitalisations, with an early study reporting a 55% reduction in high risk children and a 78% reduction in children with prematurity (without bronchopulmonary dysplasia).^19^ However, a major limitation of palivizumab is the need for monthly intramuscular injections, requiring three to six doses to maintain an adequate serum concentration throughout the RSV season,^20^ preventing many at‐risk individuals from receiving adequate protection. In contrast, nirsevimab, a long‐acting monoclonal antibody with a considerably longer half‐life, provides protection for a minimum of five months, with a single injection.^21^

## Long‐acting RSV monoclonal antibody (nirsevimab)

The safety and efficacy of nirsevimab in the prevention of severe RSV infections in infants is well recognised. Nirsevimab is now approved in over 35 countries, including Australia, with measurable impact in practical settings on reducing RSV infections.^22^

Recent clinical data from an observational study conducted in Luxembourg in the 2023 RSV season suggests that nirsevimab significantly reduced severe RSV disease in neonates.^23^ Between October and December 2023, an immunisation coverage of 84% (1277 doses for 1524 births) was achieved among neonates, with a reported 69% reduction in the number of RSV‐related hospital admissions for infants under 6 months old compared with the same period in 2022. Additionally, the number of infants under 6 months of age admitted to the paediatric intensive care unit (PICU) decreased by 68% during this same period.^23^ Similar data has emerged from Galicia, Spain, one of the pioneering regions to incorporate nirsevimab into its immunisation program. With a high coverage rate of 91.7% among 10 259 eligible infants, the program in Galicia reported an estimated 89.8% reduction in RSV‐related hospital admissions.^24^ Another study conducted in France demonstrated the significant impact of nirsevimab on PICU admissions in infants, with nirsevimab up to 80.6% effective in preventing RSV‐related PICU admissions.^25^ French surveillance data from the 2023–2024 season showed a 52.7% decrease in (all‐cause) bronchiolitis hospitalisations in infants under 3 months of age compared with 2022–2023, with the 2023–2024 season data showing the lowest number of bronchiolitis cases in this population since 2017.^26^ These findings are summarised in Box 1, highlighting the substantial reduction in PICU admissions and bronchiolitis hospitalisations following the introduction of nirsevimab. In a pooled analysis of observational real‐world studies, nirsevimab demonstrated 90.5% effectiveness in reducing RSV‐related hospitalisations at 150 days.^27^

Box 1 **Effectiveness of nirsevimab in reducing hospital and paediatric intensive care unit admissions**

**Table:** 

Study details (lead author, country, study period)	Inclusion criteria	Nirsevimab coverage	Effectiveness in reducing admissions	Safety profile
Effectiveness in reducing hospital admissions				
Ernst and colleagues^23^; Luxembourg; Sept to Dec 2023	Infants born between 1 October 2023 and 30 March 2024 with catch‐up immunisation for infants born from 1 January 2023 to 30 September 2023	84%; (1277 doses for 1524 infants)	RSV‐related LRTI hospitalisations decreased by 69% for infants under 6 months old compared with the same period in 2022 (September 2022 to December 2022)	No adverse events associated with the immunisation were reported
	Infants under 2 years of age with risk factors for severe respiratory disease			
Ares‐Gómez S and colleagues^24^; Spain; Oct 2023 to Jan 2024	Infants born between 25 September 2023 and 31 March 2024, with catch‐up immunisation for infants younger than 6 months at the start of the campaign	92%; (9408 doses for 10 259 infants)	RSV‐related LRTI hospitalisations were reduced by 89.8% in the first 3 months of the 2023–24 RSV season compared with the previous five RSV seasons (2016–17, 2017–18, 2018–19, 2019–20 and 2022–23)	Five adverse events were reported, with three categorised as severe. None of the adverse events were considered to be related to nirsevimab
	Infants under 2 years of age with risk factors for severe respiratory disease			
Effectiveness in reducing intensive care unit admission				
Paireau J and colleagues^26^; France; Sept 2023 to Jan 2024 (case‐control study; test‐negative design)	All infants with bronchiolitis (N = 288) admitted in PICU from 15 September 2023 to 31 January 2024, in metropolitan France and aged < 1 month at the start of the study or aged < 5 months at the start of the study if they had comorbidities	Over the study period, 58 infants (20%) had received nirsevimab 8 days or more before PICU admission	After adjusting for age, sex, comorbidities, prematurity and time period (high *v* low RSV circulation), nirsevimab was between 75.9% and 80.6% effective in preventing severe RSV bronchiolitis in infants requiring PICU admission	
	Infants who tested positive on multiplex PCR on nasopharyngeal swabs for RSV were classified as cases (n = 238 infants [83%]), and those who tested negative for RSV were classified as controls (n = 50 infants)			

LRTI = lower respiratory tract infections; PCR = polymerase chain reaction; PICU = paediatric intensive care unit; RSV = respiratory syncytial virus.

Moreover, the number needed to immunise (NNI) to prevent one RSV‐related lower respiratory tract infection (LRTI) hospitalisation was 25.^27^ This is similar to the NNI for rotavirus vaccines, where a deterministic transmission model in France estimated an NNI of 24.15 to 27.44 to prevent one hospitalisation.^28^ Such comparisons contextualise the potential of RSV immunisation programs to reduce hospital burden at a level similar to rotavirus vaccination. In a 2012 Australian study following the introduction of the rotavirus vaccine to the NIP, with a subsequent national vaccine coverage of 85%, a 71% reduction in rotavirus‐related gastroenteritis admissions was observed compared with the pre‐vaccination period.^29^

Reassuringly, data on safety from an infant immunisation program in the Italian region of Valle d’Aosta found that the side effects of nirsevimab were mild, with no major adverse effects reported and 89% of neonates experiencing no side effects at all.^30^ Among infants who received nirsevimab (N = 369), side effects included fever (6.5%) and localised injection site reactions (4%), with none necessitating additional medical visits.^30^ Reports of fever were based on telephone interviews at 7 and 14 days post‐administration; however, the study did not specify whether fever was measured objectively or simply parent‐reported. No instances of serious adverse effects were documented. Although this study has a small sample size, the data remain promising and offer valuable insights to inform immunisation rollouts.

The follow up of infants immunised with nirsevimab in large population groups will be important in determining the risk and severity of RSV infection in infants entering their second and subsequent RSV seasons. An observational study across 31 countries (211 sites) following children into their second RSV season reported no significant increase in medically attended RSV infections, with rates comparable to those observed during the first season.^31^ Additionally, children maintained detectable RSV antibody levels at several time points following nirsevimab administration, suggesting enduring immunity. In the MELODY and phase 2b trials, antibody levels were more than 140‐fold above baseline by Day 31, remained over 50‐fold higher at Day 151, and were still elevated compared to baseline at Day 361.^32^ However, the precise threshold for protection against future RSV infection remains uncertain, and further investigation is needed to determine the long term immunological response and whether it provides protection against future RSV infections. Nirsevimab was equally efficacious against both RSV A and B subtypes.^33^ Intensive molecular surveillance of various RSV strains has demonstrated a high degree of conservation of the nirsevimab binding site before and after the introduction of the antibody.^33^ Consequently, a single intramuscular injection of nirsevimab is anticipated to maintain high neutralisation potency, effectively neutralising over 99% of currently circulating RSV strains.^34^ Although these early data are promising, ongoing molecular surveillance programs over successive RSV seasons are imperative to identify and track any emerging polymorphisms that may lead to a reduction in the effectiveness of nirsevimab.

Wang and colleagues showed that primary RSV infection at the age of 6–23 months was associated with increased risk of asthma and wheeze compared with primary infection at the age of 0–6 months.^35^ The effects on the elderly of nirsevimab given to infants is likely to be limited, as RSV infection in older adults primarily stems from school‐age children and peers rather than infants.^36^

## Maternal vaccines

The maternal vaccine Abrysvo is another strategy for RSV prophylaxis of newborns recently added to the NIP. Maternal vaccination results in transplacental immune globulin IgG RSV antibody transfer from mother to infant,^37^ as illustrated in Box 2. In a phase 3, double‐blind, placebo‐controlled trial conducted in 18 countries (MATISSE [Maternal Immunization Study for Safety and Efficacy] study), a single dose of Abrysvo administered to 3682 pregnant women between 24 and 36 weeks of gestation was found to be 81.8% effective in preventing medically attended severe RSV disease in infants in the first 90 days of life and 69.4% effective in the first 180 days of life.^37^ Moreover, data from the trial demonstrated a favourable safety profile. Adverse events in infants, including those within the first month of birth, were similar between the vaccine and placebo groups.^38^ While a slight increase in preterm births was noted in the vaccine group, this difference was not statistically significant, and no increase in preterm birth rates was observed across high income settings.^38^ Although Abrysvo aligns well with existing maternal health care programs and provides infant protection for up to the first six months of life,^37^ there is currently limited evidence on its effectiveness beyond this period. Consideration is needed for infants requiring protection beyond the 6‐month point, such as those born prematurely or susceptible to infections after six months of age, highlighting the need for additional studies to address this gap. If infants are born prematurely, their mothers may not yet have received Abrysvo or transplacental transfer of maternal RSV antibodies may be insufficient and hence monoclonal antibodies (eg, nirsevimab) may be appropriate for these infants. Additionally, in the Northern Territory, 31% of children hospitalised with RSV‐related bronchiolitis were 6–24 months old^39^ and so these young children may benefit from a dose of nirsevimab in their second RSV season. As Abrysvo forms the foundation of Australia's national RSV immunisation program, analysing hospitalisation rates in infants beyond 6 months of age will be pivotal in assessing the vaccine's effectiveness over time. Since the current strategy primarily targets early infancy, outcomes in older infants will provide valuable data to identify any gaps in protection. This evidence will be crucial for refining the program and considering supplementary measures, such as monoclonal antibodies, to address potential vulnerabilities in populations at higher risk beyond the initial immunisation timeframe, noting that Australian jurisdictions have complementary RSV monoclonal antibody (nirsevimab) programs in 2025 for infants at increased risk.^40^, ^41^, ^42^, ^43^

Box 2 **Protective role of maternal immunisation and nirsevimab in preventing respiratory syncytial virus infection in infants aged 0–6 months**

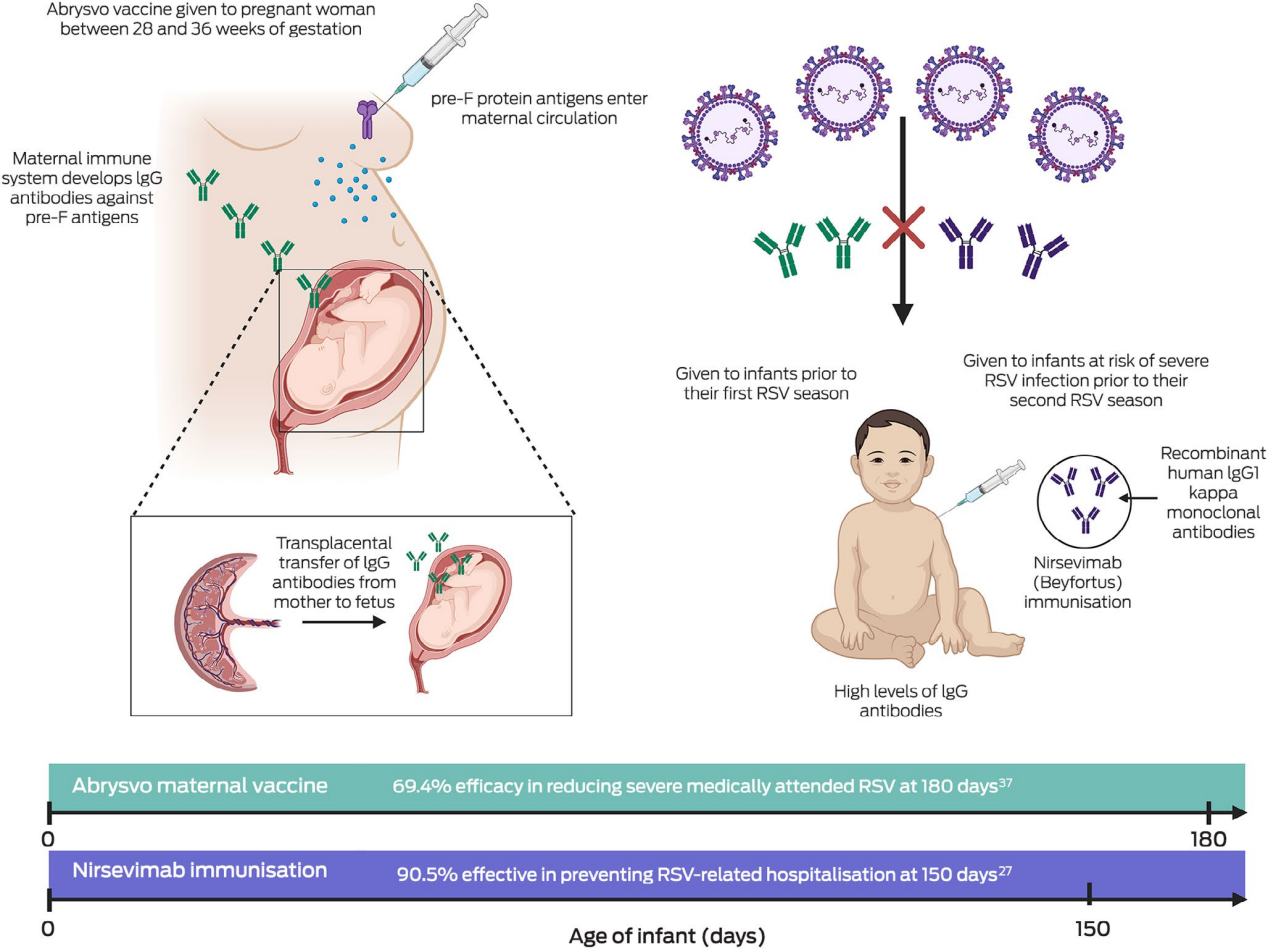

IgG = immunoglobulin; RSV = respiratory syncytial virus.

[Correction added on 4 June 2025, after first online publication: the reference citation 25 has been changed 27 and 37 respectively.]

## What else is in the pipeline?

Clesrovimab (Merck), another form of RSV prophylaxis, is also a half‐life extended human monoclonal antibody targeting the pre‐F RSV glycoprotein. Phase 3 clinical trials have recently been completed,^44^ and show that among healthy preterm and full‐term infants (birth to 1 year of age), clesrovimab reduced the incidence of RSV‐associated medically attended lower respiratory infections by 60.4% compared with a placebo.^44^ Additionally, clesrovimab lowered RSV‐related hospitalisations by 84.2% and severe RSV cases by 91.7%,^44^ highlighting its potential in preventing severe RSV infections in infants. Box 3 shows the various epitopes on the RSV pre‐F protein that palivizumab, nirsevimab and clesrovimab each bind to — sites II, Ø and IV, respectively. This variation in binding sites could become clinically relevant in time, particularly if escape mutants develop resistance to one of the antibodies. Additionally, clesrovimab has demonstrated increased availability in extravascular compartments compared with nirsevimab,^45^ a characteristic that may enhance its efficacy in preventing RSV infections.

Box 3 **Targets of respiratory syncytial virus monoclonal antibodies**

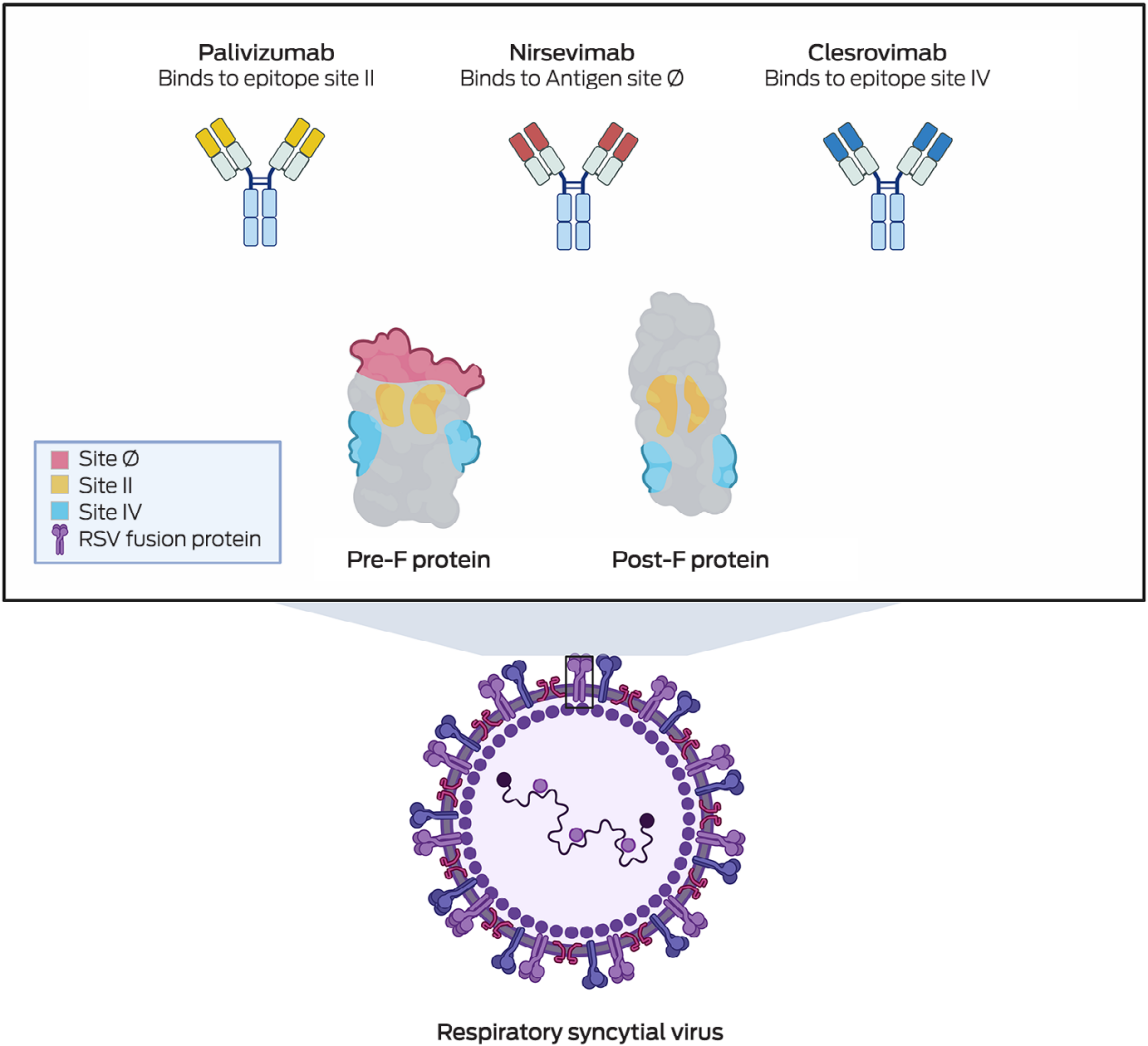

RSV = respiratory syncytial virus.

## What is the current state of play in Australia?

In 2024, nirsevimab was available to infants and children only through funded, state‐managed programs in Western Australia, Queensland and New South Wales.^46^ Preliminary impact assessments from the 2024 RSV nirsevimab immunisation programs in Western Australia and Queensland have shown encouraging outcomes. In Western Australia, where approximately 71% of eligible infants under one year of age received nirsevimab prior to or during the RSV season, RSV‐related hospital admissions were 57% lower than forecast predictions, corresponding to 505 fewer hospitalisations.^16^ In Queensland, RSV‐related hospitalisations among infants under six months of age decreased by 69% compared to the previous year, equating to one fewer hospitalisation per day in the early months of rollout, with projections suggesting that this figure could rise to three hospitalisations prevented per day by the end of the year.^17^

The economic burden of RSV is a crucial consideration when developing RSV immunoprophylaxis programs. In Australia each year, the mean per child aged under five years cost attributable to a hospital admission due to RSV disease is in excess of AUD$17 120 (2018–19),^47^ based on data from the Royal Children's Hospital, Melbourne. As a quaternary paediatric centre, this figure reflects the elevated expenses associated with intensive care unit admissions and advanced care. However, this was a single‐centre study conducted seven years ago, highlighting the need for updated and broader cost evaluations across diverse health care settings. Additional data, including results from ongoing studies, are essential to better inform cost‐effectiveness analyses for RSV prevention products. While the cost per hospitalisation may remain consistent, reducing RSV‐related hospitalisations could extensively alleviate the overall financial burden on the health care system. Until now, the cost of a routine RSV preventive program would have been prohibitive, with the average cost of a course (five doses) of palivizumab for the RSV season being AUD$9 323 (2020),^48^ a financial burden which has proven to be a key constraint to its widespread use. Currently, in the USA, the private sector cost for nirsevimab is USD$519.75 (~AUD$827; exchange rate from January 2025 [USD$1 = AUD$1.59]) per dose for both 50 mg and 100 mg doses,^49^ while in European countries, the mean price was estimated at €339 (~AUD$567).^50^ In comparison, the private sector cost of Abrysvo in the USA is USD$295 (~AUD$469) per dose, representing a potentially more cost‐effective strategy for broad population‐level protection.

Abrysvo offers a more practical alternative to palivizumab and is available on the Australian private market at $331.99 per dose.^50^ Nirsevimab, while also included in the national strategy, is not currently available for private purchase and is distributed exclusively through government‐funded programs. The inclusion of Abrysvo in the NIP highlights the critical role of public funding in ensuring equitable and sustainable access to RSV prevention.

In 2025, all Australian jurisdictions have an RSV Mother and Infant Protection Program (MIPP), with the maternal RSV vaccine funded under the NIP commencing on 3 February 2025^51^ and complementary monoclonal antibody (nirsevimab) programs funded by individual Australian state and territory governments. The program aims to reduce RSV‐related morbidity through the administration of a single dose of Abrysvo between 28 and 36 weeks’ gestation, thereby providing protection for infants against RSV. In addition to this, eligible infants, including infants born to mothers who did not receive Abrysvo during pregnancy or those with high risk conditions, will have access to the long‐acting monoclonal antibody nirsevimab at no cost, ensuring equitable protection for all infants at risk of RSV disease. Program monitoring and evaluation, including cost‐effectiveness modelling, from the 2025 RSV MIPPs will help guide future decisions regarding the optimal combination of maternal vaccination and nirsevimab. These studies, which will incorporate Australian‐specific data, will evaluate factors such as cost‐effectiveness, timing, geographic variability and logistical considerations in relation to administration. Although formal study announcements are still pending, these efforts are likely to form part of broader national evaluations accompanying RSV immunisation implementation. Surveillance activities, such as those captured by FluCAN and other systems listed by the Australian Centre for Disease Control,^52^ are also contributing to ongoing RSV monitoring and may inform these assessments.

An evidence‐based approach will be essential to ensure appropriate allocation of resources, tailoring programs to the specific needs of higher risk populations while maximising the economic and health benefits across Australia.

In addition to preventing severe RSV infections, it is also important to consider how RSV immunoprophylaxis programs may reduce recurrent wheeze and asthma following RSV infections in children. A comprehensive birth cohort study conducted in South Africa revealed that hospitalisation due to RSV infection was associated with a higher prevalence of recurrent wheeze compared with milder cases of RSV managed in the community.^53^ Although the observed adverse effects on lung function may be a consequence of RSV‐related LRTIs, it is also plausible that these effects reflect an underlying predisposition to both symptomatic RSV infection and long term respiratory conditions, such as asthma. However, recurrent LRTIs were also significantly more prevalent following an initial RSV infection,^53^ underscoring the potential long term impact of RSV on respiratory health during early childhood. Another recent study published by Rosas‐Salazar and colleagues demonstrated in a birth cohort of more than 1700 children that avoiding RSV infection during infancy could prevent up to 15% of childhood asthma cases.^54^ Well designed longitudinal studies to determine how early RSV prevention impacts future lung function and risk of asthma are needed.

To ensure the success of future RSV preventive programs in Australia, the timing, geographical variation and associated logistics of service delivery must also be considered. Since July 2021, RSV has been classified as a notifiable disease under the National Notifiable Diseases Surveillance System,^2^ offering crucial data on the temporal and geographical variations in RSV infections across Australia. In light of the national program, the timing of maternal RSV immunisation (administered between 28 and 36 weeks gestation) and the accurate recording of this information in the Australian Immunisation Register (AIR) will be crucial for monitoring vaccine uptake and ensuring continuity of care. As the service delivery for the maternal vaccine and infant monoclonal antibody may be managed by different health care providers, it is essential to capture this information on the AIR to enable comprehensive tracking of immunisation receipt across both strategies. Integrating data from both the maternal vaccine and nirsevimab, has already been initiated by participating states in 2024, but will require ongoing monitoring in future years to ensure immunisations are recorded and to optimise program effectiveness and assess long term respiratory outcomes.

Another important consideration is parental acceptance towards the new RSV preventives. A 2024 national survey that included both future and current parents of children aged under 5 years in Australia assessed current attitudes towards future RSV prevention strategies. Among the 1992 participants, 93.4% of future and 81.4% of current parents exhibited a high level of acceptance towards infant RSV immunisation before receiving information on RSV.^55^ Similarly, 79.7% of pregnant or planning parents and 86.9% of future parents (no other children) were willing to receive a maternal RSV vaccine during pregnancy.^55^ Although a 2019 study in Melbourne^56^ found that only 17% of pregnant women had heard of RSV, almost half (48%) of the participants were born outside Australia, highlighting the need for broad multi‐language programs to explain the risks and benefits of RSV prevention strategies. Reassuringly, both studies^55^, ^56^ underscored the importance of education, as parents who were initially unaware of RSV demonstrated increased acceptance towards RSV preventives after being provided with relevant information. Understanding consumer decision making will be critical for policy makers and health care providers, particularly as the maternal vaccine landscape has three vaccines (influenza, pertussis and coronavirus disease 2019) currently recommended and included on NIP during pregnancy.

It is vital to prioritise the health of First Nations communities, who are disproportionately affected by severe RSV infections. In Central Australia, where First Nations Australians represent more than 41.8% of the population,^57^ the annual incidence of RSV infection in hospitalised children was found to be 29.6 per 1000 in First Nations children under the age of 2 years. This is nearly three times the 10.9 per 1000 incidence of their non‐First Nations counterparts.^58^ However, these figures may underestimate the true burden of RSV in First Nations communities. A WA study that developed a prediction model to estimate the burden of RSV in hospitalised children showed that laboratory‐confirmed RSV cases were likely underestimated, particularly among infants aged under 3 months. Children in this age group accounted for 22.4% of those tested but one in three RSV‐positive admissions and were found to have significantly higher odds of RSV positivity compared to older children. Notably, higher hospitalisation rates were observed among children from rural and remote regions, highlighting the increased susceptibility to RSV infection of rural and remote‐dwelling children.^59^ It is therefore essential to prioritise distribution of RSV preventives to these populations.

## Conclusion

The landscape of RSV prevention in Australia rapidly evolved in 2024 with the introduction of two new prophylactic measures to protect infants: immunisation with long‐acting monoclonal antibody nirsevimab and maternal RSV vaccine Abrysvo. Maternal vaccination offers the advantage of protecting infants from birth through the passive transfer of antibodies, while nirsevimab provides direct and extended protection of infants during times of peak RSV activity.

Cost‐effectiveness remains a pivotal consideration in the implementation of national RSV preventive program in Australia. Both nirsevimab and Abrysvo have demonstrated substantial potential in reducing hospitalisation rates and mitigating long term health care costs associated with RSV infections in infants. Future pricing strategies, informed by ongoing research and real‐world efficacy data, will be critical to ensure the sustainability of both preventives for a broader population.

The 2025 federal and complementary jurisdictional programs for RSV prevention underscore a commitment to addressing the burden of RSV. Successful implementation will hinge on the timing and location of administration, addressing uptake and acceptance and ensuring accessibility to special risk groups including First Nations communities and other groups at increased risk. Continued surveillance, monitoring and evaluation will be essential to monitor the impact of these important interventions and adjust future RSV prevention strategies as indicated.

There is also an ongoing need to protect other age groups for which there are no preventives currently available. These include children older than 8 months (or 2 years for high‐risk) and young adults. The recent halting of clinical trials using mRNA vaccines in infants due to a potential safety signal highlights the ongoing challenges in RSV vaccine development in children.^60^

## Open access

Open access publishing facilitated by The University of Melbourne, as part of the Wiley – The University of Melbourne agreement via the Council of Australian University Librarians.

## Competing interests

Jane Tuckerman and Danielle Wurzel are named on an investigator‐led project grant sponsored by GlaxoSmithKline. Danielle Wurzel has received consultancy fees from Merck Sharp and Dohme and Praxhub, which have been directed to a research fund. Nigel Crawford is the Chair of the Australian Therapeutic Advisory Group on Immunisation (ATAGI), where he provides evidence‐based health advice to government on vaccines and immunisation science, to guide public health policy, procedures and clinical use of the vaccines. All other authors have no conflicts of interest to declare.

## Provenance

Not commissioned; externally peer reviewed.
